# Exosomes from adipose-derived stem cells regulate M1/M2 macrophage phenotypic polarization to promote bone healing via miR-451a/MIF

**DOI:** 10.1186/s13287-022-02823-1

**Published:** 2022-04-08

**Authors:** Rui Li, Dize Li, Huanan Wang, Kaiwen Chen, Si Wang, Jie Xu, Ping Ji

**Affiliations:** 1 Department of Pediatric Dentistry, The College of Stomatology, Chongqing Medical University, No. 426, North Songshi Road, Yubei District, Chongqing, 401147 People’s Republic of China; 2 Department of Oral and Maxillofacial Surgery, Chongqing Key Laboratory of Oral Diseases and Biomedical Sciences, Chongqing, People’s Republic of China; 3 Chongqing Municipal Key Laboratory of Oral Biomedical Engineering of Higher Education, Chongqing, People’s Republic of China; 4 Key State Laboratory of Fine Chemicals, School of Bioengineering, Dalian University of Technology, Dalian, 116023 People’s Republic of China

**Keywords:** Exosomes, Adipose-derived stem cells, Macrophages, Bone healing, MiRNA

## Abstract

**Objectives:**

Bone defects caused by diseases and trauma are usually accompanied by inflammation, and the implantation of biomaterials as a common repair method has also been found to cause inflammatory reactions, which affect bone metabolism and new bone formation. This study investigated whether exosomes from adipose-derived stem cells (ADSC-Exos) plays an immunomodulatory role in traumatic bone defects and elucidated the underlying mechanisms.

**Methods:**

ADSC-Exos were loaded by a biomaterial named gelatine nanoparticles (GNPs), physical and chemical properties were analysed by zeta potential, surface topography and rheology. A rat model of skull defect was used for our in vivo studies, and micro-CT and histological staining were used to analyse histological changes in the bone defect area. RT-qPCR and western blotting were performed to verify that ADSC-Exos could regulate M1/M2 macrophage polarization. MicroRNA (miRNA) array analysis was conducted to determine the miRNA expression profiles of ADSC-Exos. After macrophages were treated with a miR-451a mimic, miR-451a inhibitor and ISO-1, the relative expression of genes and proteins was measured by RT-qPCR and western blotting.

**Results:**

In vivo, micro-CT and histological staining showed that exosome-loaded GNPs (GNP-Exos) hydrogel, with good biocompatibility and strong mechanical adaptability, exhibited immunomodulatory effect mainly by regulating macrophage immunity and promoting bone tissue healing. Immunofluorescence further indicated that ADSC-Exos reduced M1 marker (iNOS) expression and increased M2 marker (CD206) expression. Moreover, in vitro studies, western blotting and RT-qPCR showed that ADSC-Exos inhibited M1 macrophage marker expression and upregulated M2 macrophage marker expression. MiR-451a was enriched in ADSC-Exos and targeted macrophage migration inhibitory factor (MIF). Macrophages treated with the miR-451a mimic showed lower expression of M1 markers. In contrast, miR-451a inhibitor treatment upregulated the expression of M1 markers and downregulated the expression of M2 markers, while ISO-1 (a MIF inhibitor) treatment upregulated miR-451a expression and downregulated M1 macrophage marker expression.

**Conclusion:**

GNP-Exos can effectively regulate bone immune metabolism and further promote bone healing partly through immune regulation of miR-451a, which may provide a therapeutic direction for bone repair.

**Supplementary Information:**

The online version contains supplementary material available at 10.1186/s13287-022-02823-1.

## Introduction

Craniomaxillofacial bone defects due to the influences of age, trauma and various diseases severely affect the physiological function and facial morphology of patients [1]. However, effective repair of these defects remains a clinical problem. At present, autogenous bone transplantation is the most commonly used method in the clinic [2, 3], but this method has considerable limitations, such as the large defect area and discomfort at the donor site. In addition, immune rejection and infection have become the main causes of repair failure after allogeneic bone transplantation [4, 5]. Therefore, new solutions to effectively promote bone healing and regeneration are urgently needed. In recent years, with the rapid development of material technology, it is more and more widely used in bone tissue repair, which is a good repair scheme [6, 7], but it is found that biomaterials, as a foreign substance, usually induce immune response and inflammation, leading to the failure of implantation [8–10]. Studies have shown that the immune system is closely related to the skeletal system and that these systems cooperate with each other [11, 12]. Immune cells and their metabolites participate in the regulation of bone deposition and bone strength by affecting the activity of osteoblasts, osteoclasts and osteocytes [13]. Bone defects caused by trauma and tumours are usually accompanied by features of peripheral inflammation and immune imbalance, including acute ischemia and hypoxia, release of pro-inflammatory and anti-inflammatory factors, abnormal cell metabolism, etc. Macrophages are important components of the innate immune system and play a regulatory role in inflammation [14, 15]. M1 macrophages are considered to have a pro-inflammatory phenotype, while M2 macrophages have an anti-inflammatory phenotype and can secrete anti-inflammatory factors, induce progenitor cells and promote growth factor release. Promoting M2 macrophage phenotype polarization can effectively promote bone angiogenesis and bone healing [16–18]. So we considered about whether we can promote successful bone healing by regulating immunity.

Recently, bone tissue engineering (BTE) combined stem cells, growth factors and biomaterial scaffolds to obtain biological substitutes that can reshape craniomaxillofacial morphology, which can better make up for the defects of single application [19–21]. Adipose-derived mesenchymal stem cells (ADSCs) are commonly used in BTE, besides they can differentiate into a variety of cell types to improve the functional recovery of different types of damaged tissues [22–25], their immune regulation has attracted increasing attention. These cells play therapeutic roles in many immune diseases by secreting anti-inflammatory cytokines and growth factors that counteract inflammatory factors and can effectively regulate the immune response caused by biomaterial implantation [26–28]. ADSCs are easy to obtain through minimally invasive surgery, with the advantages of high yield, low invasion and high proliferation rate; thus, they are considered to have excellent prospects for future clinical applications [29, 30]. An increasing number of studies have indicated that combinations of ADSCs and biomaterials can play important roles in tissue repair [31–33]. In BTE, when used as carriers of the stem cells, various materials can give appropriate physical, chemical and mechanical stimulation to the cells, providing a favourable environment for maintaining stem cell stability and promoting stem cell function [34, 35]. However, stem cell therapy generally has the disadvantages of low inoculation rate, cell abscission, limited number of adherent cells and low biological activity [36, 37]. Even the application of biomaterials cannot significantly improve its effective bioavailability [19]. At present, ADSCs are produced primarily with the dish culture method, which hinders the isolation and transportation of large numbers of available stem cells. In addition, due to the low survival rate and cell retention rate, cells may not be successfully implanted into the receptor [38]. Nowadays, existing studies have shown that the therapeutic effects of stem cells are mediated mostly through paracrine signalling, mainly are exosomes [39–41]. Due to the above limitations of cell therapy, so more and more studies began to focus on exosomes.

The exosomes secreted by stem cells are lipid double-layer binding vesicles with a diameter of 50–150 nm that contain multiple bioactive molecules (proteins, nucleic acids, lipids and RNA molecules), which can be transferred to target cells through ligand–receptor interactions, endocytosis or direct membrane fusion [42–44]. Thus, exosomes play roles in intercellular communication, immune regulation and cell signal transduction, ADSC-Exos can also effectively participate in the human immune response and play an active role through immune regulation [45–48]. Therefore, research on immunomodulation in bone repair involving exosomes has increased, and new therapies that use ADSC-Exos to inhibit periosteal inflammation and promote bone healing are being developed [49]. Although many advantages of exosomes have been proved for tissue engineering. It remains challenging for the in situ stay of exosomes as well as the stable long-term delivery. Gelatine nanoparticles (GNPs) have been used for tissue engineering because of their high biocompatibility and obvious mechanical adaptability [50]. As a partial derivative of collagen, gelatine also promotes cell adhesion by upregulating host cell recognizing and integrin attachment [51]. In the previous study, GNPs hydrogel has been proved excellent rheological characteristic, while the ideal shear thinning and self-healing property endows it great injectability to fill irregular bone defect sites [52, 53]. Aiming to the inadequate strength and initial burst release of GNPs, the electronegative exosomes can electrostatically assemble with GNPs and are expected to improve the mechanical properties and more stable drug delivery.

Therefore, in our experiments, we used an injectable GNPs hydrogel, a good nano-structure hydrogel with self-healing ability and viscoelasticity, to load ADSC-Exos into a rat skull defect model. We observed the effects of GNP-Exos on bone healing and repair and explored the immunomodulatory mechanism in vitro. This study demonstrates that ADSC-Exos can effectively regulate the immune response around bone and provide a new treatment method to improve bone healing.

## Materials and methods

### Ethics statement

The study was approved by the Ethical Committee of Chongqing Medical University (027), and written informed consent was obtained from all the participants. All collected tissue samples were processed in accordance with the Declaration of Helsinki.

### Extraction and identification of ADSCs

The subcutaneous adipose tissue collection during the operation has been informed and agreed by the patient. ADSCs were isolated and cultured as previously described [54]. Briefly, the collected adipose tissue was cleaned to remove the surrounding fascia and blood vessels and cut into small pieces and treated with 0.1% collagenase I (Sigma-Aldrich, St. Louis, MO, USA) for 60 min at 37 °C. The digested tissue was washed with α-MEM (HyClone, USA) containing 10% FBS (Bioind, Biological Industry, Israel) and then centrifuged at 1200×*g* for 5 min. The cell pellets were washed, resuspended in α-MEM (HyClone, USA) supplemented with 10% FBS and 1% penicillin/streptomycin solution, and cultured at 37 °C and 5% CO2. To evaluate the multilineage differentiation potential of ADSCs, ADSCs were subjected to flow cytometry to assess their phenotypic characteristics. CD44, CD105, CD90, CD31, CD19, CD34 and HLA-DR levels were detected. ADSCs, as a kind of MSCs with multi-differentiation potential, were cultured in adipogenic differentiation medium, stained using oil red O after 14 days or cultured in osteogenic differentiation medium and stained with alkaline phosphatase after 9 days.

### Isolation of ADSC-conditioned medium (CM) and exosomes

CM from ADSCs was collected as follows: ADSCs (passage 2–4) were seeded in 10-cm culture dishes at 37 °C with 5% CO2 until reaching 80–90% confluence. Then, the culture medium was removed from each dish, and the dishes were washed with PBS, after which ADSCs were cultured in serum-free medium for 48 h. The supernatant was collected and centrifuged at 1500 rpm for 10 min to remove cell debris, and ADSC-CM was finally obtained. Exosomes were isolated using ultracentrifugation as follows: after ADSCs (passage 2–4)were cultured in serum-free medium for 48 h, culture supernatants collected from ADSCs were centrifuged at increasing speeds of 300×*g* for 10 min, 2000×*g* for 10 min, and 10,000×*g* for 30 min to remove cell debris, after which the collected supernatants were ultracentrifuged at 100,000×*g* for 70 min in an ultracentrifuge (Hitachi, CP100NX, Japan) to obtain primary exosomes. Finally, the pellet was washed with PBS and centrifuged at 100,000×*g* for 70 min to purify the exosomes. We resuspended the exosome pellet in 100 µl sterile PBS and stored it at − 80 °C.

### Characterization of ADSC-Exos

For particle size determination, nanoparticle tracking analysis (NTA) and transmission electron microscopy (TEM) were performed in accordance with the protocols. Briefly, the collected exosomes were fixed with 1% glutaraldehyde at 4 °C overnight. After washing, the vesicles were loaded onto formvar/carbon-coated nickel TEM grids and incubated for 30 min. After removing the excess fluid, our samples were then stained with aqueous phosphotungstic acid for 60 s and finally imaged by TEM. The size and concentration of vesicles were determined by a NanoSight tracking analysis system (Brookhaven Instruments Corp, USA).

### Manufacturing of the exosome-loaded GNPs (GNP-Exos) hydrogel

We prepared the exosome-loading hydrogel according to the methods in our previous study [55]. Briefly, 1 ml of PBS containing 0.8 mg of ADSC-Exos was immediately loaded into a Luer-lock medical syringe and subsequently mixed with the contents of another Luer-lock syringe containing 0.1 g of freeze-dried GNPs powder by repetitive extrusion of the mixtures to obtain the GNP-Exos hydrogel.

### Characterization: zeta potential, surface topography, and exosome distribution

In order to evaluate the electrostatic assembly of GNPs and exosomes, a Zetasizer Nano ZSP (Malvern Panalytical, China) was used to evaluate the zeta potential values of the GNPs, ADSC-Exos and GNP-Exos. The surface topographies of the GNP-Exos samples were investigated with field-emission scanning electron microscopy (FE-SEM), while laser confocal microscopy (LSCM, TCS.SP8, Leica, Germany) was used to detect the exosomes distribution.

### Rheology

We used a Discovery Hybrid Rheometer (Anton Paar, Austria) to test the rheologic properties of the GNP-Exos. The operating gap distance was set to 1000 μm. The viscoelastic properties of the GNP-Exos were characterized by oscillatory frequency sweep (0.1 to 10 rad/s at a constant strain of 0.5%) and strain sweep (0.1 to 100% strain at a constant frequency of 1 Hz). The self-healing capacity of the GNP-Exos was assessed according to the recovery of G’ and G’’ of the gel network.

### Release behaviour of ADSC-Exos

After fluorescent labelling of the exosomes, 1 ml of PBS containing 0.8 mg of labelled ADSC-Exos was mixed with 0.1 g of GNPs using a Luer-lock syringe. Then, the hydrogel was placed in 5 ml of PBS at 4 °C. All the samples were protected from light. Each day, 1 ml of PBS containing released ADSC-Exos was collected, after which 1 ml of fresh PBS was added into the release system. The fluorescence intensity (585/601 nm) of the collected samples was measured using an EnSpire Multimode Plate Reader (PerkinElmer, USA), and the actual released volume was calculated based on the standard curve for ADSC-Exos.

### Animal and surgical protocols

The animal experiments were performed following the ARRIVE guidelines, and all Sprague–Dawley (SD) rats were purchased from the Experimental Animal Center of Chongqing Medical University. The animals were provided unlimited access to food and water and were housed under a 12 h light/dark cycle at 23 ± 2 °C. In our experiment, we used injectable GNPs hydrogel-loaded exosomes into skull defects in rats. The surgery was carried out under sterilized conditions, and 8 × 104 IU/day penicillin was administered to each animal postoperatively. 36 SD rats were divided into three groups: the control, GNPs, and GNP-Exos groups. All rats received anaesthesia by sodium pentobarbital injection (30 mg/kg), and a linear incision was made on the median area of the calvaria in the anterior–posterior direction. The periosteum was detached in opposite directions, followed by the creation of 5-mm-diameter bone defects on each side of the parietal region using a trepan. Afterwards, pure gelatine-based hydrogel was injected and shaped in the skull defects of rats in the GNPs group, while hydrogel-loaded exosomes were implanted in the skull defects of rats in the GNP-Exos group. For the control group, nothing was administered to the defect sites, and the wound was sutured directly.

### Microcomputed tomography (micro-CT)

After animal killing at 4 and 8 weeks, the calvarial bone of rats was collected and fixed in 4% paraformaldehyde overnight. Then, micro-CT (SANCO Medical AG, Switzerland) was used to evaluate new bone formation in defect sites. The parameters were determined to be 70 kV and 112 μA with a thickness of 0.048 mm per slice in a medium-resolution mode 1024 reconstruction matrix and 200 ms integration time. A total of 315–543 Hounsfield units were selected as the threshold values of bone. In addition, the bone volume fraction (BV/TV), trabecular number (Tb.N), trabecular thickness (Tb.Th), trabecular separation (Tb.Sp) and bone formation rate were calculated using ImageJ, and the volume of interest (VOI) was defined as the cylinder at the centre of each defect.

### Histological staining

To further evaluate osteogenesis in calvarial bone defects with implanted exosome-loaded GNPs, standard haematoxylin and eosin (H&E) staining and aniline blue staining were performed. After paraffin embedding, the fixed samples were sliced into sections at 8 μm. For aniline blue staining, the sections were successively placed into a 5% phosphotungstic acid solution and 1% aniline blue solution.

### Immunofluorescence staining

To evaluate the immunomodulation of GNP-Exos in vivo, double immunofluorescence staining was carried out with primary antibodies against F4/80 (macrophage marker) and iNOS (M1 marker) or CD206 (M2 marker). Three non-adjacent slices of each sample were analysed with 3 samples in each group.

### Cell culture

The human monocyte cell line U937 was purchased from the Chinese Academy of Sciences (Shanghai, China) and cultured in RPMI 1640 medium (HyClone, USA) supplemented with 1% penicillin–streptomycin and 10% FBS (Biological Industries, Israel). U937 cells were stimulated with 100 ng/ml phorbol 12-myristate-13-acetate (Sigma-Aldrich) for 24 h. To induce the M1 phenotype, the cells were further incubated with 2 µg/ml lipopolysaccharide (LPS) (Sigma-Aldrich) and 50 ng/ml IFN-γ (Sinobiological, China) in fresh medium. Subcutaneous adipose tissue was collected from four patients during the surgical procedure and immediately immersed in sterile PBS.

### Treatment of macrophages with ADSC-CM and ADSC-Exos

U937 cells were stimulated with 100 ng/ml phorbol 12-myristate-13-acetate (Sigma-Aldrich) for 24 h to obtain macrophages. To induce the M1 phenotype, the cells were further incubated with 2 µg/ml lipopolysaccharide (LPS) (Sigma-Aldrich) and 50 ng/ml IFN-γ (Sinobiological, China) in fresh medium. Macrophages were pretreated with ADSC-CM (diluted 1:1 with α-MEM containing 10% foetal bovine serum) and ADSC-Exos (200 µg/ml) for 2 h followed by stimulation with LPS and IFN-γ. For gene expression analysis, samples were harvested after 24 h; for protein expression analysis, samples were harvested at 48 h; and for the phosphokinase array, samples were harvested at 30 min.

### Fluorescent labelling of exosomes: endocytosis experiments

The collected exosomes were labelled with a PKH67 Linker Kit (Sigma-Aldrich, USA) according to the manufacturer’s protocol, and PBS was used to replace exosomes as a control. According to the above experimental steps, after 24 h of cell seeding, the labelled exosomes and the control (PBS) were added to the culture medium and cultured at 37 °C for 0.5 h and 1 h; after removing the original medium, the cells were washed three times with PBS, 10 min each time, and fixed in 4% neutral buffered formalin. Nuclei were stained with 4′,6-diamino-2′-phenylindole (DAPI). Finally, images were captured using an Olympus microscope (Olympus, Japan).

### Real-time RT-qPCR

For gene expression analysis, the cells were harvested and lysed in RNAiso plus (TaKaRa, Japan), after which RNA was quantified with NanoDrop (Thermo Scientific, USA) and reverse-transcribed using the PrimeScript™ RT Reagent Kit (TaKaRa, Japan) to obtain cDNA. For miR-451a expression analysis, after RNA isolation, first-strand cDNA was synthesized via a miRNA First Strand Synthesis Kit (Sangon Biotech, China) according to the manufacturer’s instructions. RT-PCR was performed with TB Green to quantify mRNA levels on a Bio-Rad real-time PCR system (CFXConnect, USA), which were calculated by the 2 − ΔΔCt method. The expression levels of target genes were normalized to the expression levels of the control housekeeping gene β-actin. MiR-100 expression was normalized to that of U6. The primers for target genes are included in Additional file 1: Table S1.

### Western blot assay

Cells were lysed and centrifuged at 12,000 rpm at 4 °C following the addition of a protease inhibitor. Protein concentrations in purified exosomes and cells were quantified using the Enhanced BCA Protein Assay Kit (Beyotime, China). For western blot analysis, 30 μg of cell lysates or SHED-Exo lysates was loaded for each sample, separated by 10% SDS-PAGE and transferred to PVDF membranes. The primary antibodies used to assay protein expression were against calnexin (1:1000, CST, USA), CD9 (1:800, ProteinTech, USA), CD63 (1:1000, Abcam, USA), CD86 (1:800, Bioss, China), CD206 (1:1000, Abcam, USA) and iNOS (1:1000, Abcam, USA), and β-actin (1:5000, Bioss, China) was used as the loading control (Bioss, China). Images were captured and analysed with a computer programme (ImageJ, USA).

### Transfection of miRNA-451a

After U937 cells were stimulated with 100 ng/ml phorbol 12-myristate-13-acetate (Sigma-Aldrich) for 24 h, the obtained macrophages were seeded on culture slides. The macrophages were transfected with 50 nM miR-451a mimic, 100 nM miR-451a inhibitor, or the corresponding control oligonucleotide (miR-451a mimic negative control (NC) or miR-451a inhibitor NC) using Entranster™-R4000 transfection reagent (Engreen Biosystem Co., Ltd., China) in 2 ml of RPMI 1640 medium according to the manufacturer’s instructions. Six hours post-incubation, the medium was removed, and the cells were washed with PBS and transfected for 24–48 h. The sequences were as follows: hsa-miR-451a mimic (sense, 5′-AAACCGUUACCAUUACUGAGUU-3′; antisense, 5′-CACAAGUUCGGAUCUACGGGUU-3′), hsa-miR-451a inhibitor (sense, 5′-AACUCAGUAAUGGUAACGGUUU-3′), mimic NC (sense, 5′UUGUACUACACAAAAGUACUG-3′; antisense, 5′-GUACUUUUGUGUAGUACAAUU-3′), and inhibitor NC (sense, 5′-CAGUACUUUUGUGUAGUACAA-3′). All oligos were synthesized by Sangon Biotech (Shanghai, China).

### Treatment with a macrophage migration inhibitory factor (MIF) inhibitor

Induced cells were incubated with the MIF inhibitor ISO-1 (10 µM) (Selleck, USA) for 2 h and then stimulated with LPS and INF-γ for 24 h followed by mRNA expression analysis.

### Statistical analysis

GraphPad Prism 7.0 (GraphPad Software, USA) was employed to performed statistical analyses. Data are expressed as the mean ± SEM. Statistical differences were evaluated using Student’s t test or one-way ANOVA with Tukey’s post hoc test for multigroup comparisons, and P values < 0.05 were considered significant.

## Results

### Characterization of ADSCs and ADSC-Exos

**Fig. 1:**
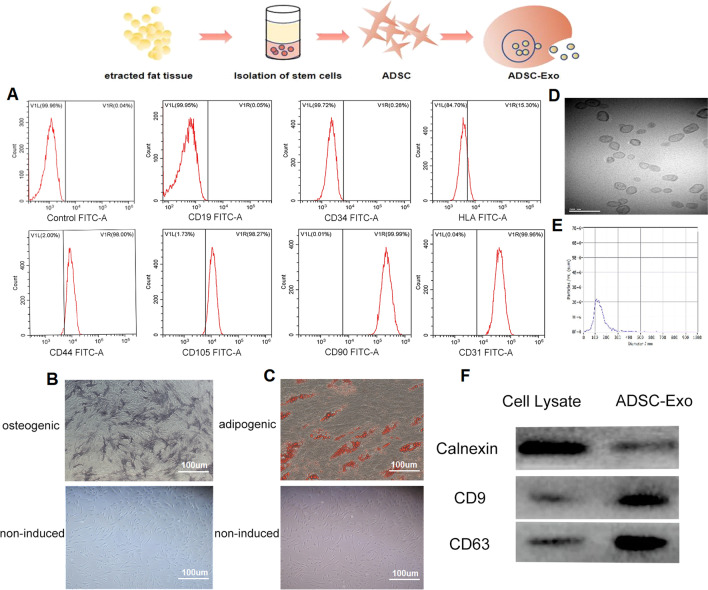
Characterization of ADSCs and ADSC-Exos.** A** Flow cytometric analysis of the expression of ADSC surface markers. CD44, CD105, CD90 and CD31 are positive and CD19, CD34, and HLA-DR are negative. **B** The differentiation potential of ADSCs assessed by oil red O staining after 14 days of adipogenic induction and alkaline phosphatase staining after 9 days of osteogenic induction.** C** TEM showing ADSC-Exo morphology. Scale bars indicate 200 nm.** D** Particle size of ADSC-Exos was measured by Zetasizer Nano.** E** Surface exosomal markers of ADSC-Exos (CD63, CD9) were quantified by western blotting

In this study, ADSCs were isolated and their typical morphology was confirmed under a light microscope. These cells were found to be positive for CD44, CD105, CD90, and CD31 but did not express the haematopoietic markers CD19, CD34, or HLA-DR by flow cytometry (Fig. 1A). Multi-directional differentiation potential of ADSCs was evaluated by staining with alkaline phosphatase after osteogenic induction (Fig. 1B) and oil red O after adipogenic induction (Fig. 1C), we observed that non-induced ADSCs could not be successfully stained, while osteogenic- and adipogenic-induced ADSCs showed obvious positive staining characteristics. Electron micrographs showed the characteristic cup-shaped morphology, and most of the exosomes had a characteristic morphology with sizes ranging from 80 to 130 nm (Fig. 1D). NTA revealed that the size distribution of exosomes was approximately 120 nm, in agreement with the characteristics of exosomes (80–130 nm; Fig. 1E). In addition, western blot analysis of exosome lysates showed positive expression of the surface exosomal marker proteins CD9 and CD63 compared to ADSC lysates (Fig. 1F). These data indicated that we successfully collected ADSCs and ADSC-Exo particles.

### Characterization of the GNP-Exos hydrogel

**Fig. 2:**
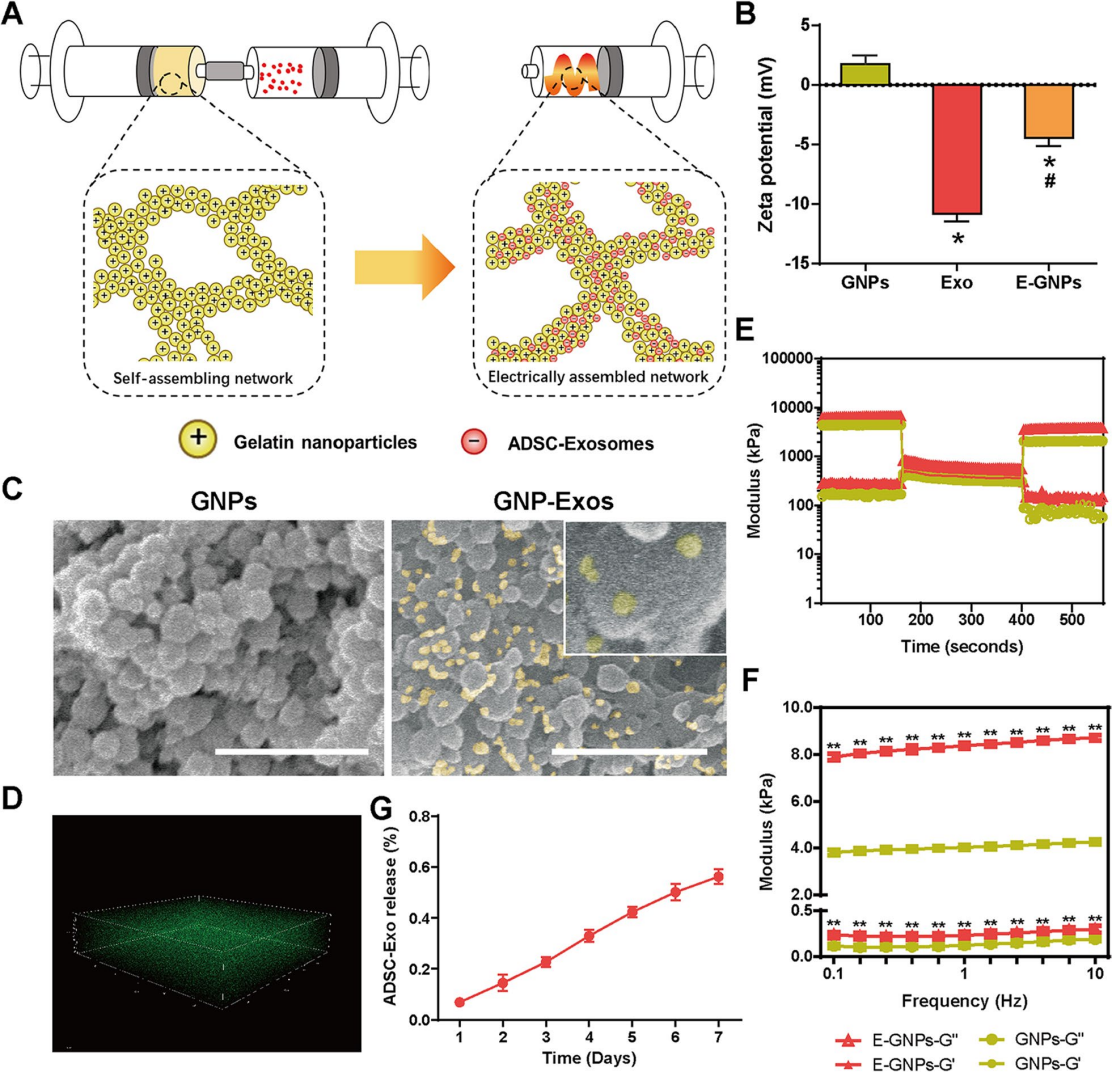
GNP-Exos material characterization.** A** Schech showing the electrostatically assemble of GNPs and DSC-Exos.** B** Zeta potential of GNPs, ADSC-Exos and GNPs-Exo.** C**,** D** SEM and LSCM showing distribution of ADSC-Exos in GNPs hydrogel in 2D and 3D approach (Scale bar = 1 μm).** E**,** F** rheology of GNPs-Exo including the viscoelastic properties and self-healing capacity.** G** Release behavior of ADSC-Exos in PBS. * P < 0.05, ** P < 0.01 using one-way ANOVA and Student-Newman-Keuls test

Given the opposite charge characteristics of GNPs and ADSC-Exos, the improved physical properties of the electrostatically assembled network were expected (Fig. 2A). The zeta potential of GNPs was 1.75 ± 0.75 mV; however, after the GNPs were mixed with ADSC-Exos (− 10.80 ± 0.65 mV), the resulting GNP-Exos exhibited a decreased zeta potential of − 4.47 ± 0.66 mV (Fig. 2B), which confirmed that electrostatic assembly occurred. In addition, FE-SEM and LSCM showed evenly distributed ADSC-Exos in the GNPs network (Fig. 2C, D). The cohesive interactions among nanoparticles were also proven by the significantly increased modulus and ideal self-healing behaviour (Fig. 2E, F). After 100% strain was applied for 3 min, the G’ modulus of GNP-Exos recovered to at least 53.2%, showing the self-healing potential of the electrostatically assembled network. Next, the release of exosomes from GNP-Exos was detected (Fig. 2G). As they met the prerequisite of cohesive interactions, the GNP-Exos exhibited ideal controlled release potential. Stable release of ADSC-Exos was detected for at least 7 days, and for each day, the daily release was in the range of 9.81–16.36%. More importantly, the initial burst release could be advantageously supressed, and approximately zero-order release was observed (R2 = 0.99). It is well established that the burst drug release was a limitation for many existing drug carriers for they cannot completely control the delivery speed. The zero-order release means drug release rate rarely change by the time of delivery, suggesting GNP-EXOs obviously avoided the disadvantageously burst release.

### ADSC-Exos promote the bone healing of skull defects in rats

**Fig. 3:**
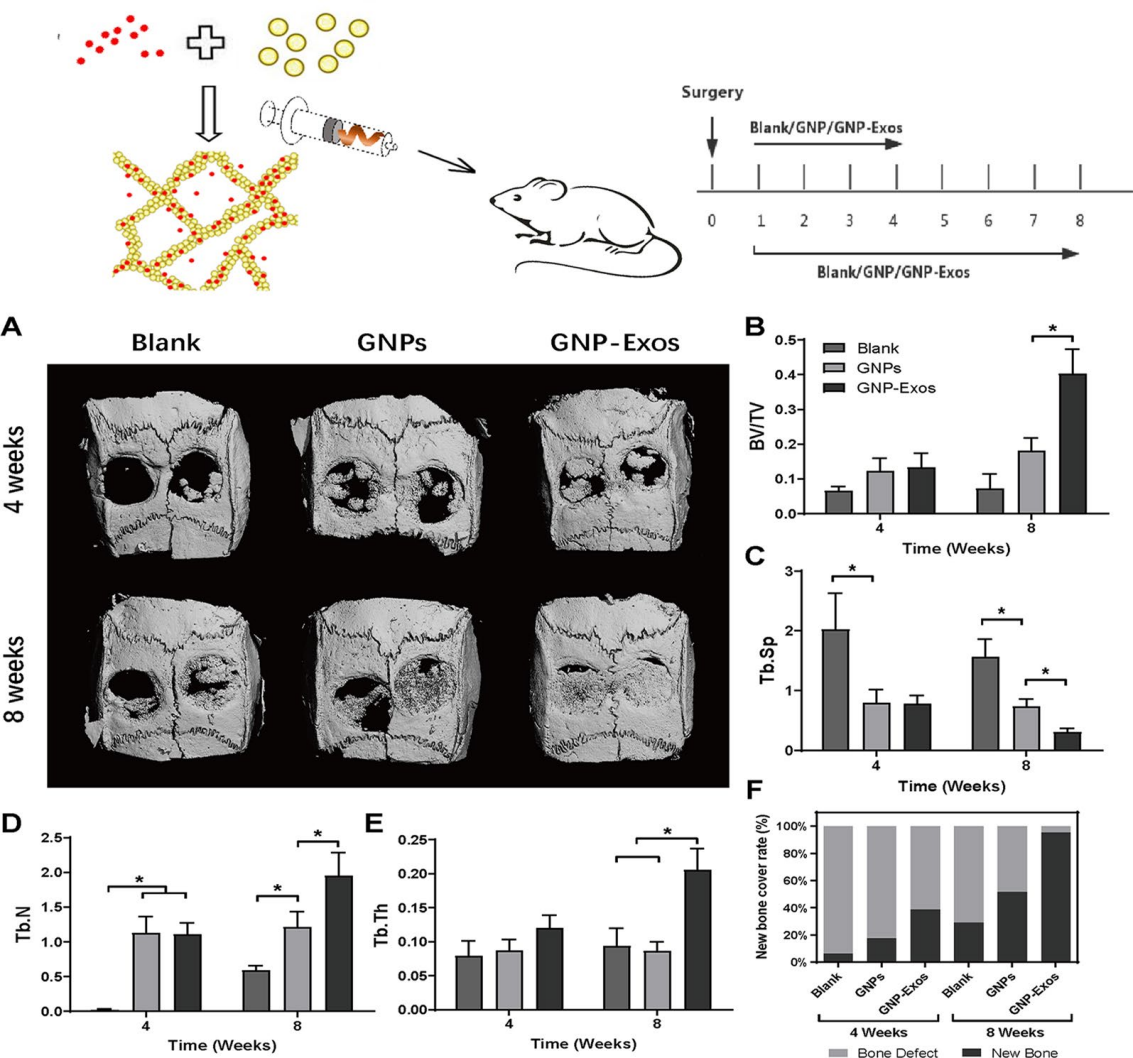
The micro-CT measurement and semi-quantitative analysis of SD rats treated with pure gelatine nanoparticle hydrogel (denoted as GNPs) and exosome-loaded gelatine nanoparticle hydrogel (denoted as GNPs-Exo) implanted in calvarial bone defects. **A** Micro-CT imaging of calvarial bone at 4 and 8 weeks postoperatively.** B**–**F** The new bone formation rate and bone formation-related parameters (BV/TV, Tb.N, Tb.Th, Tb.Sp) were calculated. * P < 0.05, ** P < 0.01 using one-way ANOVA and Student-Newman-Keuls test

**Fig. 4:**
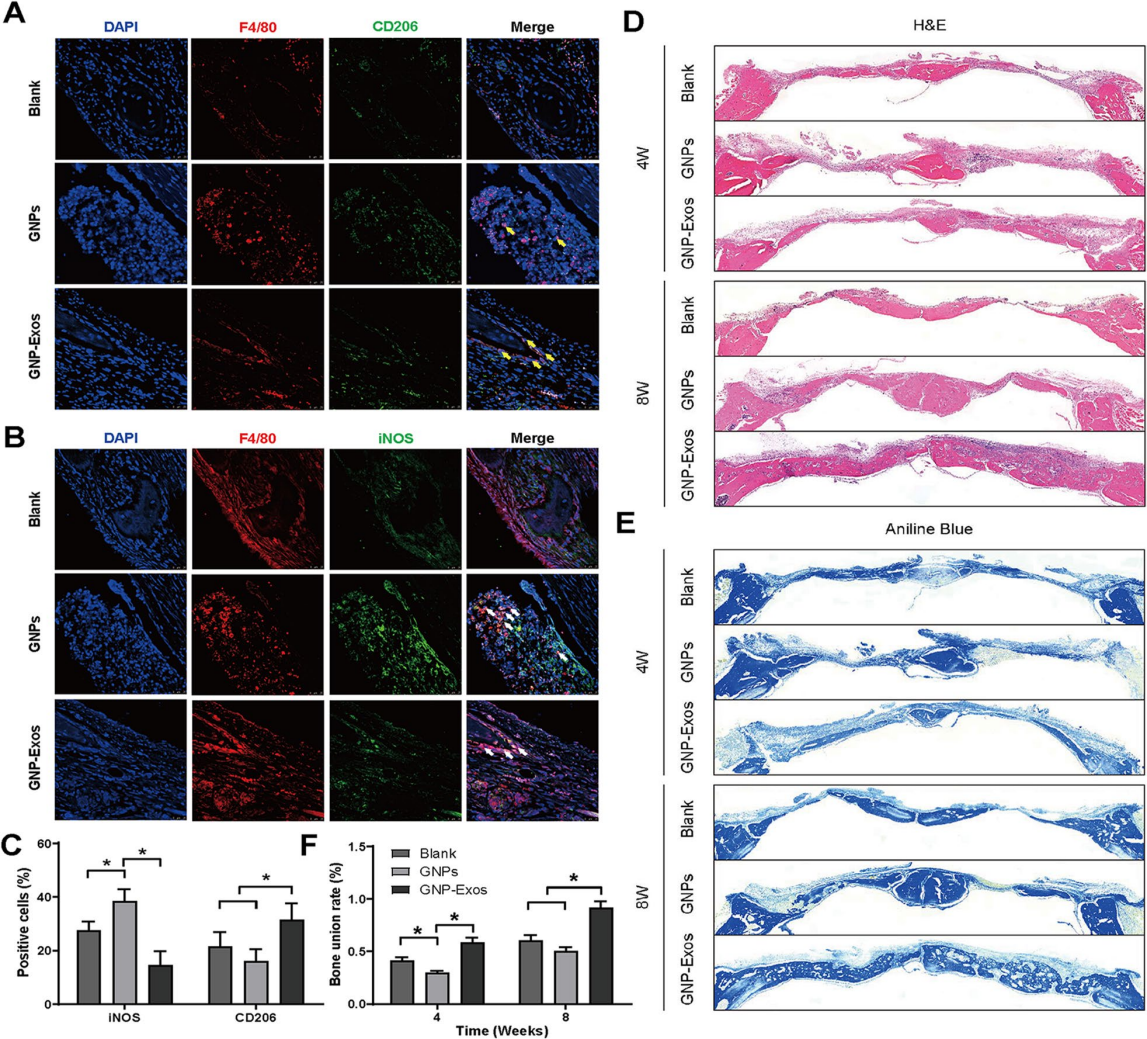
The effect of ADSC-Exos on bone defect in rats. Histological staining of calvarial bone defects in rats using H&E staining (**D**) and aniline blue (**E**).** F** Semi-quantitative analysis of bone union by histological staining. Immunofluorescence staining of rats’ calvarial bone implanted with GNP hydrogel or exosome-loaded GNP hydrogel. iNOS and CD206 were used as markers of M1 and M2 macrophages and double stained with F4/80 (a marker of macrophages) at 4 and 8 weeks after implantation. **A** and** B** are iNOS and CD206 immunofluorescence staining of bone defect sections respectively.** C** Semi-quantitative statistics of iNOS and CD206 positive cells. The significance levels are indicated: ns: not significant, *P < 0.05

For the prepared GNPs and GNP-Exos, we first conducted routine biosafety tests in animal experiments (Additional file 2: Figure S1), and results showed that the materials used in the study have good biocompatibility. As shown in Fig. 3A, a model of skull defects in rats was successfully established. GNPs partially promoted new bone formation in the calvarial defect area at 4 and 8 weeks after surgery. Although no significant difference between the BV/TV of the GNPs and control groups was observed, a notable decrease in Tb.Sp and increase in Tb.N were found in the GNPs group (Fig. 3C–E). Notably, GNP-Exos treatment strongly boosted calvarial bone formation compared to the blank and GNPs groups. The BV/TV of GNP-Exos-treated bone defects was increased by fourfold, while other parameters of osteogenesis indicated a higher quality of newly formed bone (Fig. 3B). More importantly, as shown by the new bone formation rate (Fig. 3F), GNP-Exos promoted 95% bone coverage at 8 weeks. Therefore, we believe that the sustained release of exosomes from GNPs strongly promotes osteogenesis. Histological staining was performed to further evaluate osteogenesis in bone defects. As shown by aniline blue staining, at 4 weeks, a large amount of fibrous tissue was observed in defect sites with no or GNPs treatment. In comparison, bone tissue under the periosteum was generated from the broken ends of calvarial bone defects after treatment with GNP-Exos. At 8 weeks, GNPs loaded with exosomes induced significant formation of woven bones that almost covered the whole defect area and were in close union to the original bones (Fig. 4D–F).

### ADSC-Exos enhanced bone healing, which may be related to the change in the phenotypic distribution of macrophages around bone injuries

As shown in Fig. 4A–C, GNPs caused higher iNOS expression than the control, what may be related to the immune response caused by the material entering the body as a foreign matter. However, the application of GNP-Exos significantly decreased the expression of iNOS and increased the expression of CD206. INOS and CD206 were used as markers of M1 and M2 macrophages, respectively, so we speculated that GNP-Exos induce M1-to-M2 polarization to further established an anti-inflammatory microenvironment. In general, compared with GNP treatment group and blank control group, the exosomes released from GNPs hydrogel could notably regulate M2 polarization and therefore influence downstream osteogenic cell functions to consequently achieve ideal bone repair.

### ADSC-Exos promote the polarization of LPS/IFN-γ-treated M1 macrophages towards the M2 phenotype

**Fig. 5:**
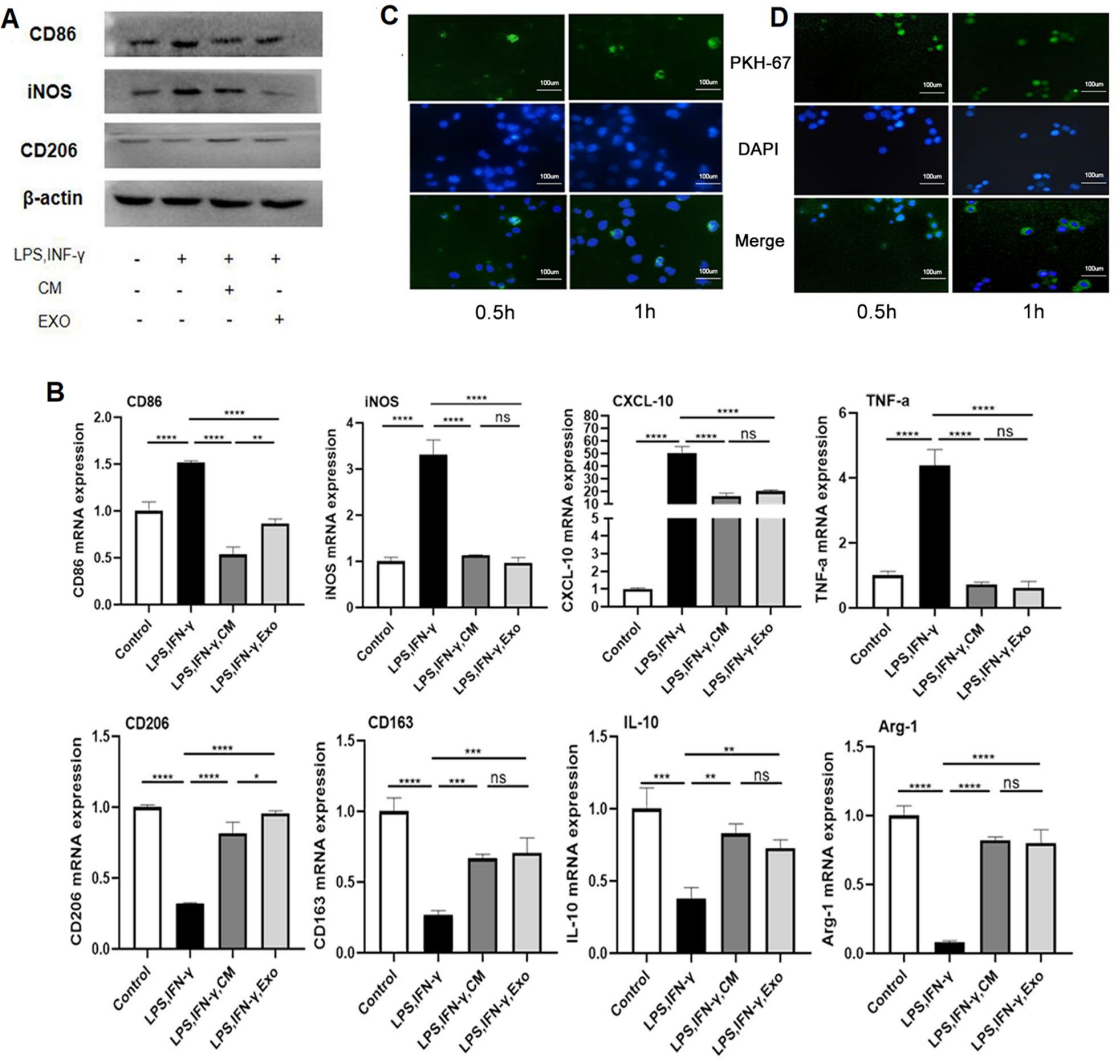
ADSC-Exos promote the polarization of LPS/IFN-γ-treated M1 macrophages towards the M2 phenotype. U937-derived M1 macrophages were pretreated with ADSC-CM and ADSC-Exos followed by stimulation with LPS/IFN-γ.** A** The protein levels of the M1 macrophage markers iNOS and CD86 and the M2 macrophage marker CD206 were analysed by western blotting.** B** M1 macrophage markers and M2 macrophage markers were detected at the mRNA level by RT-qPCR. ADSC-Exos (**C**) and released from GNPs’ADSC-Exos (**D**) were labelled with PKH67 (green), cocultured with macrophages for 0.5 h and 1 h, and fluorescence signals were examined by fluorescence microscopy. Data are representative of at least two independent experiments. The significance levels are indicated: ns: not significant, *P < 0.05, **P < 0.01, ***P < 0.001, ****P < 0.0001

As shown above, it was found that ADSC-Exos may promote the polarization of M1-M2 macrophages in the bone defect experiment. Therefore, we hope to further verify the results in vitro and study its possible internal mechanism. Unfortunately, our current experimental technology cannot successfully extract the macrophage population found in the degenerating or regenerating bone tissue. But macrophages in bone tissue or any other tissue are all derived from initial macrophages, so we switched to a monocyte cell line (U937) to investigate the mechanism of ADSC-Exo effects in vitro. To elucidate the roles of ADSC-Exos in M1 and M2 macrophage crosstalk, we pretreated macrophages with ADSC-CM and ADSC-Exos (200 µg/ml) for 2 h, followed by stimulation with LPS and IFN-γ. The protein levels of the M1 macrophage markers iNOS and CD86 and the M2 macrophage marker CD206 were analysed by western blotting, and results showed that macrophages treated with LPS and IFN-γ highly expressed CD86, iNOS, while ADSC-CM and ADSC-Exo pretreatment decreased their expression and increased CD206 expression (Fig. 5A). Furthermore, the expression of macrophage related factors at the gene level was detected, consistent with the western blot results, ADSC-CM and ADSC-Exo could reduce CD86, iNOS, TNF-α and CXCL-10(M1 markers) mRNA expression and increase CD206 and IL-10(M2 markers) mRNA expression (Fig. 5B), these results further confirm our hypothesis. In addition, ADSC-Exos were labelled with PKH67 and incubated with macrophages; Fig. 5C reveals the uptake of exosomes by macrophages at 0.5 h and 1 h and green fluorescence increased with the incubation time. Similarly, the exosomes released from GNPs also successfully entry into cells and concentrate near the nucleus after coculture with macrophages, which indicated exosomes released from GNP still have vitality. But it is also found that the fluorescence intensity of exosomes is weak and the morphology is changed compared with that of simple exosomes (Fig. 5D).

### ADSC-Exos promote M1-to-M2 macrophage polarization through miR-451a

**Fig. 6:**
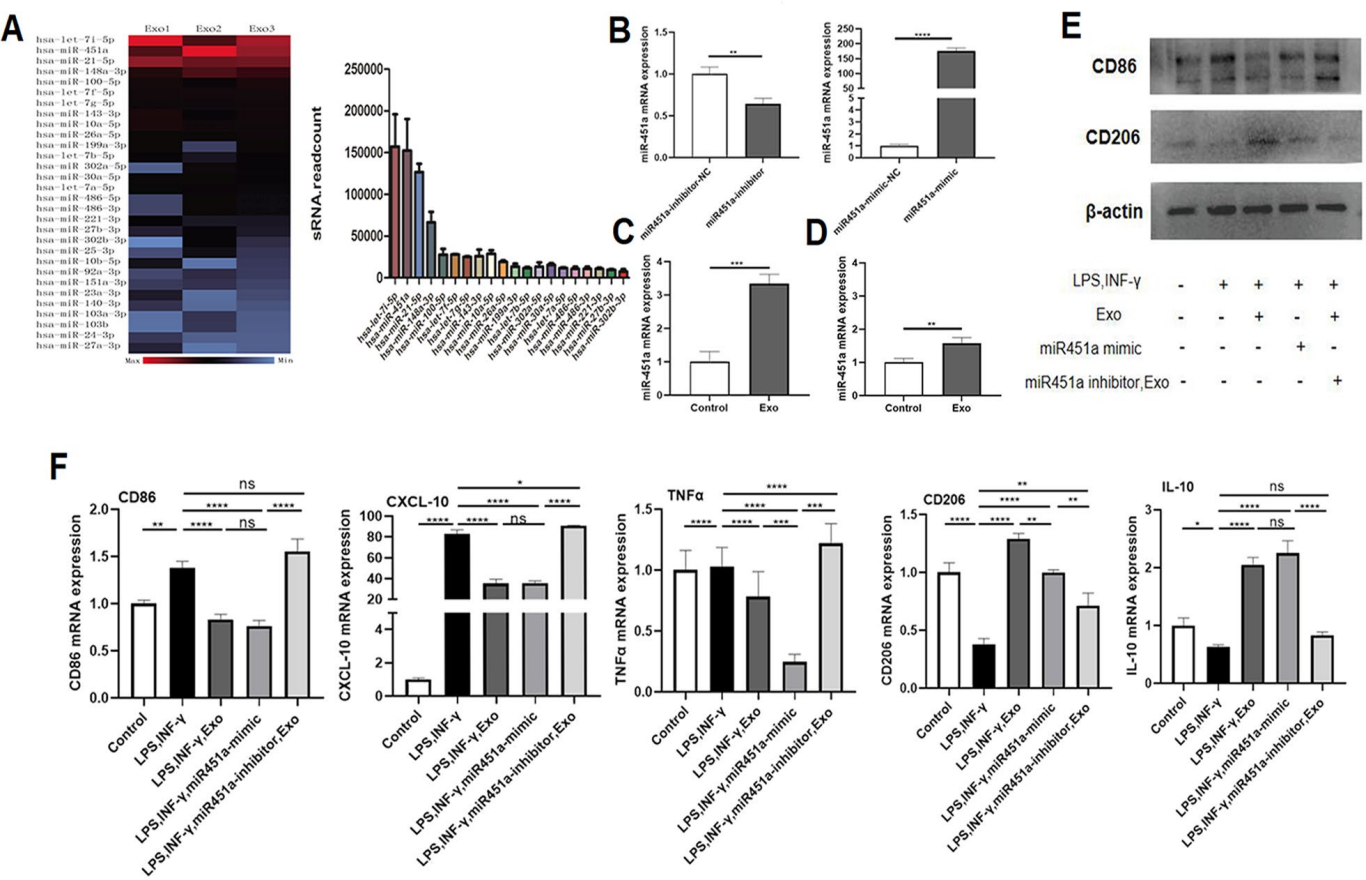
MiR-451a, a highly expressed miRNA in ADSC-Exos, promotes macrophage M1 to M2 polarization.** A** Through miRNA microarray analysis, we obtained the top 20 differentially expressed miRNAs in ADSC-Exos. Red indicates high expression and blue indicates low expression.** B** Macrophages were transfected with the miR-451a-mimic, miR-451a-inhibitor, miR-451a-mimic-NC, and miR-451a-inhibitor-NC; miR-451a levels were determined by RT-qPCR.** C** miR-451a expression in ADSC-Exo-treated macrophages was detected by RT-qPCR. **D** miR-451a expression in exosomes released from GNPs treated macrophages was detected by RT-qPCR. Statistical analysis was performed using Student’s t test.** E** The protein levels of the M1 macrophage marker CD86 and the M2 macrophage marker CD206 were analysed by western blotting.** F** mRNA expression of M1-macrophage markers and M2-macrophage markers was detected by RT-qPCR. Statistical analysis was performed using one-way ANOVA followed by Tukey’s test. Data with P values < 0.05 were considered significant. *P < 0.05, **P < 0.01, ***P < 0.001, ****P < 0.001. All data are expressed as the mean ± SEM

Through microarray analysis, we determined the expression profile of miRNAs in ADSC-Exos and identified the top 20 miRNAs with the most significant difference in expression. MiR-451a was more abundant in ADSC-Exos than other miRNAs (Fig. 6A). In order to explore the role of miR-451a, we upregulated and downregulated miR-451a expression in macrophages through treating with miR-451a mimic and miR-45ia inhibitor firstly (Fig. 6B). At the same time, it is found that the expression of miR-451a increased in macrographs after ADSC-Exo treatment compared with the control treatment (Fig. 6C). Similarly, exosomes released from GNPs were cocultured with macrophages also upregulated the expression of miR-451a (Fig. 6D). As shown in Fig. 6E, western blotting results found that the expression levels of CD86 were significantly decreased in ADSC-Exo-treated macrophages and in miR-451a-overexpressing macrophages, whereas they were increased in the miR-451a inhibitor group. Conversely, the expression levels of CD206 showed the opposite trends, which indicated miR-451a inhibitor can resist the effect of ADSC-Exo. Furthermore, the mRNA expression of other M1 and M2 macrophage-related factors was detected by RT-qPCR, consistent with western blot results, results showed that CD86, TNF-α and CXCL-10 (M1 marker) mRNA expression were significantly decreased in ADSC-Exo-treated macrophages and in miR-451a-overexpressing macrophages, whereas they were increased in the miR-451a inhibitor group. And the mRNA expression levels of CD206 and IL-10 (M2 marker) showed the opposite trends (Fig. 6F).

### MiR-451a promoted macrophage M1-to-M2 polarization via repression of MIF

**Fig. 7:**
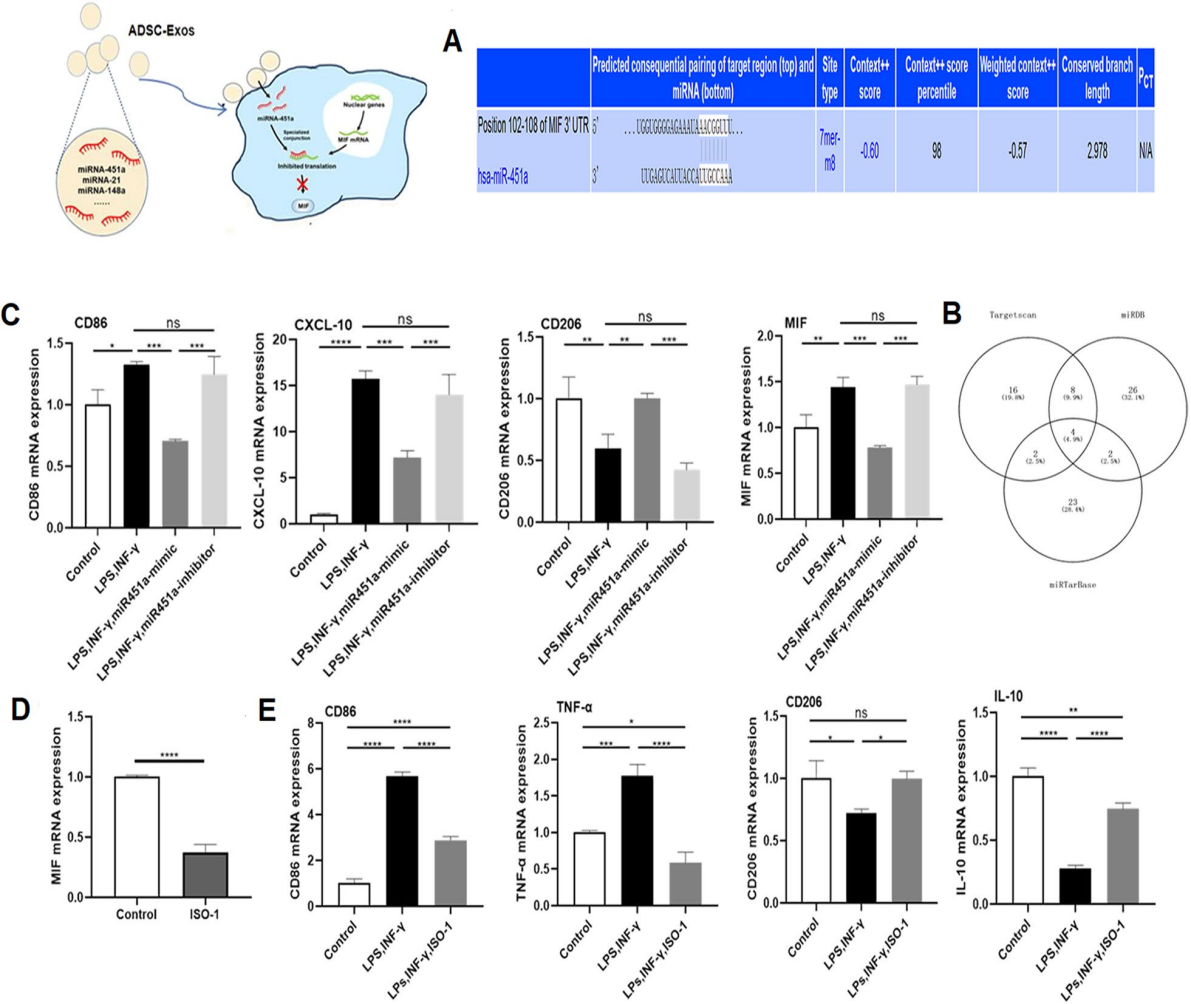
MiR-451a promotes macrophage M1 to M2 polarization and suppresses inflammation via repression of macrophage migration inhibitory factor (MIF).** A**,** B** MIF, as a possible target gene of miRNA-451a, was revealed to bind to miRNA-451a directly by bioinformatics software analyses (Targetscan,miRDB,miRTarBase).** C** The mRNA levels of M1 macrophage markers and M2 macrophage markers were detected by RT-qPCR.** D** Macrophages were treated with rapamycin (10 nM) to inhibit MIF, and MIF expression levels were quantified by RT-qPCR. Statistical analysis was performed by Student’s t test.** E** The expression of M1 macrophage markers and M2 macrophage markers were quantified by RT-qPCR. Data are presented as the mean ± SEM. Data with P values < 0.05 were considered significant; *P < 0.05, **P < 0.01, ***P < 0.001, ****P < 0.001

Our data showed that ADSC-Exos play a role through miR-451a. Next, bioinformatics analysis using Targetscan, miRDB, miRTarBase revealed that MIF may be its downstream target (Fig. 7A, B). As shown in Fig. 7C, RT-qPCR revealed that MIF expression was upregulated when the cells were stimulated with LPS/IFN-γ but downregulated after transfection with miR-451a. To further explore the role of MIF in macrophage phenotypic polarization, ISO-1 (a MIF inhibitor) was used. ISO-1 inhibited the expression of MIF compared with the control treatment (Fig. 7D). When MIF was inhibited, the expression of CD206 and IL-10 was increased, while the expression of CD86 and TNF-α was decreased (Fig. 7E).

## Discussion

**Scheme 1:**
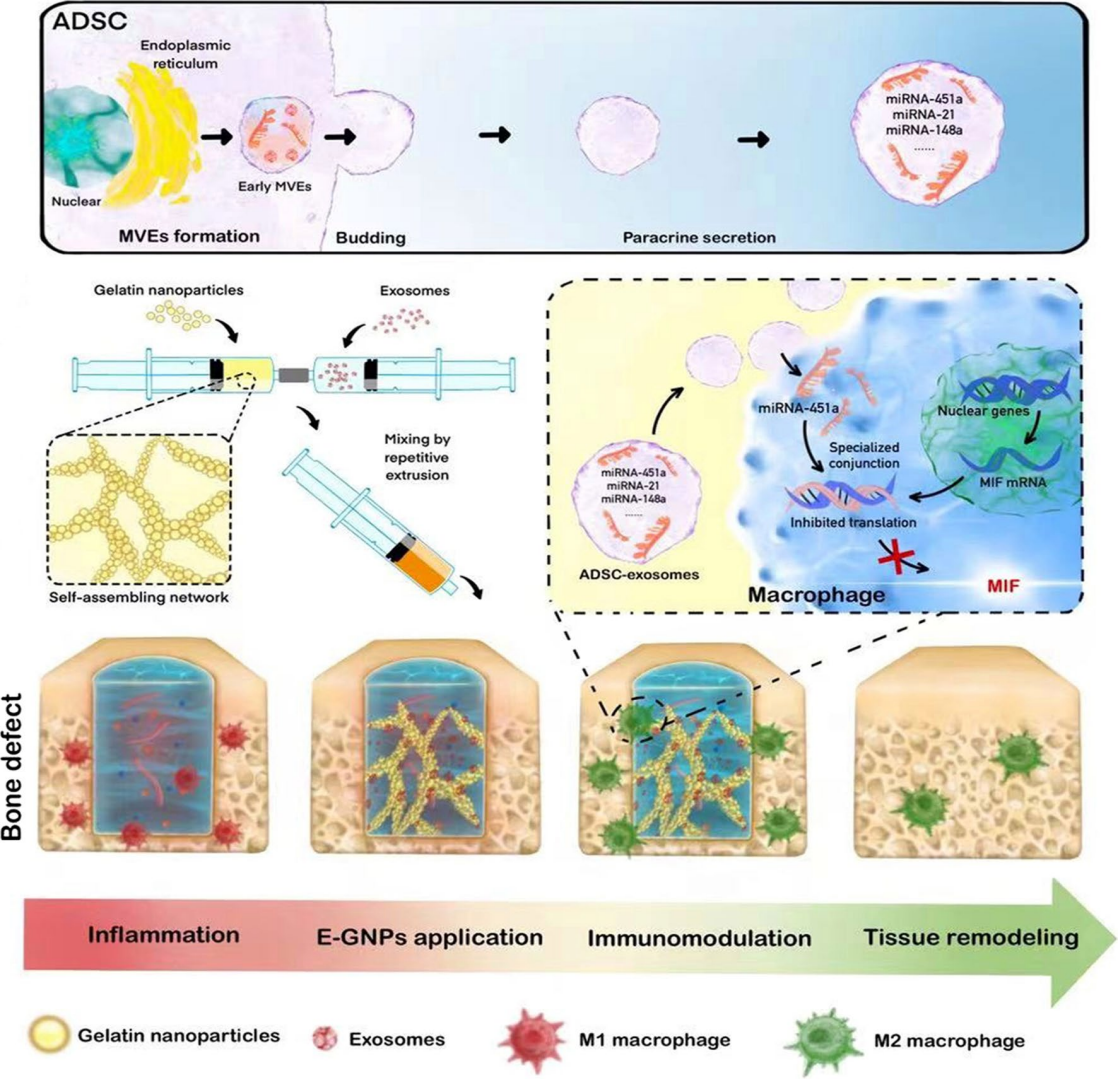
Schematic illustration of exosome-loaded GNPs (GNP-Exos) hydrogel promote bone healing by immunomodulating macrophage polarization

In this study, in vivo and in vitro experiments showed that GNP-Exos can promote the transformation of M1 macrophages to M2 macrophages, thus inhibiting the inflammatory response around bone defects and accelerating bone healing. In addition, we found that mir-451a was highly expressed in ADSC-Exos and participated in immunoregulation as an effective anti-inflammatory component. This study elucidates the role and potential mechanism of ADSC-Exos in M1/M2 macrophage polarization and provides relevant evidence for future research on the potential therapeutic value of ADSC-Exos in immunoregulation and bone healing (Scheme 1).

As mentioned above, exosomes must be transported to appropriate locations to play their roles in vivo, and how to ensure that they persist stably and function in the target locations is a question that must be addressed. The ideal hydrogel for osteogenesis should have appropriate mechanical strength and adaptability. In this study, the exosome-loading hydrogel had a G’ value almost one order of magnitude higher than that of the pure GNPs hydrogel due to the electrostatic attraction between the exosomes and GNPs (Fig. 1B), which formed a stable network. As shown in the rheological self-healing test, rapid recovery of the G’ value of the GNP-Exos hydrogel (55.5 ± 4.2%) occurred within approximately 4 s after severe destruction (produced via application of 100% shear strain for 240 s). This property makes the GNP-Exos gel adaptable to the local complexity at a defect. In addition, hydrogels should act as bridges for bioactive exosome loading and delivery to help prevent burst release as well as drug loss during storage and transportation. The GNPs were electrostatically attracted to the exosomes and bound to them, which advantageously increased the drug loading rate and achieved stable drug release for at least 1 week. Therefore, the therapeutic period could ideally cover the initial immune response and subsequent early osteogenesis. It is worth noting that Bio-Oss bone grafts are usually used as positive control of osteogenic treatment. In comparison with existing Bio-Oss application in rats’ calvarial bone defects at the same time points [56], the hard tissue proportions of GNP-EXOs (40.27% ± 7.05%) was higher than the Bio-Oss application (39.95% ± 6.81%), indicating the ideal osteogenesis of GNP-EXOs. In the rat skull defect model, the injectable GNPs hydrogel successfully loaded exosomes and stabilized them in the skull defects. The results showed that during bone healing, the numbers of M1 macrophages decreased, while the numbers of M2 macrophages increased. Furthermore, in order to explore whether the exosomes released from GNPs retained activity and function, we collected exosomes from GNPs and used them to treat macrophages. Immunofluorescence microscopy showed that ADSC-Exos (Fig. 5C) successfully entered the cells and became distributed around the nucleus, but the fluorescence intensity of the exosomes was significantly decreased, and the exosomes released by the materials were different in size and morphology. In order to further prove that the exosomes released from GNPs still have biological effects, we collected the exosomes and co-cultured them with macrophages to detect the expression of mir451a. The results (Fig. 6D) showed that the expression of mir451a in macrophages treated with exosomes was significantly higher than that in the control group.

A lot of research in the past have found that ADSC-Exos can effectively participate in the immune response and play an active role through immune regulation [57–59]. Recent studies have shown that ADSC-Exos improve myocardial injury after myocardial infarction and reduce adipose inflammation and obesity by regulating the phenotypic polarization of macrophages [47, 60]. In our study, we found that when only GNPs hydrogel was injected around the bone defect, a positive effect on bone healing was observed compared with that in the control group, but it was also found that there were many M1-type macrophages. However, ADSC-Exos treatment significantly reduced the number of M1 macrophages and increased the number of M2 macrophages. In addition, in vitro experiments showed that ADSC-Exos promoted the polarization of M1 macrophages to M2 macrophages. Consistent with our previous hypothesis, the ADSC-Exos were able to accelerate bone healing by inhibiting the inflammatory response around the bone defect. However, the exact mechanism underlying these functions is still unclear. Previous studies have proven that miRNAs contained in exosomes are an important component of exosomes, playing a significant role in regulating cell growth and metabolism [61]. For example, Yingjie Lu found that ADSC-Exos can promote angiogenesis through miR-486-5p, thus accelerating wound healing. Zhao et al. proved that MSC-Exos attenuate myocardial ischaemia–reperfusion injury through miR-182-regulated macrophage polarization [62, 63]. Therefore, we analysed the miRNA expression profile of ADSC-Exos, and the results showed that miR-451a was highly expressed in exosomes. In order to further verify the findings, we treated macrophages with exosomes. PCR showed that the expression of mir-451a was significantly higher (by approximately threefold) in the exosome-treated group than in the control group. Similarly, the expression of mir-451a was higher in macrophages treated with exosomes released by GNPs than in control macrophages.

At present, several studies have demonstrated the role of miR-451 in cancer: overexpression of miR-451 inhibits the proliferation and induces the apoptosis of osteosarcoma cells and inhibits the proliferation, invasion and migration of neuroblastoma [64, 65]. Some scholars have also found that miR-451 protects against cardiomyocyte anoxia/reoxygenation injury by inhibiting high mobility group box 1 expression [66]. However, the role of miR-451 in immune regulation has not been clarified. In order to test our hypothesis that ADSC-Exos exert an immunomodulatory effect through mir-451a, we transfected mir-451a analogues and mir-451a inhibitors into macrophages and induced differentiation of M1 macrophages with LPS and IFN-γ. The results (Fig. 7C) showed that ADSC-Exos promoted macrophage polarization partly through mir-451a. Next, we searched for the target gene of miR-451a with multiple target gene prediction websites and ultimately determined that MIF was a possible target gene. This finding is consistent with other recent findings that mir-451 specifically combines with the MIF mRNA 3'UTR, which directly regulates the expression of MIF [67, 68]. As an endocrine immune molecule, MIF can limit the activity of macrophages and participate in immune regulation in vivo. After inducing M2 macrophages, Mariana’s group found that inhibition of MIF significantly reduced the expression of macrophage M2 markers. In addition, MIF inhibitors can inhibit the activation of macrophages and the expression of inflammatory factors, such as NO, TNF-α and IL-6, which can significantly improve arthritis and articular cartilage injury in rats [69, 70]. In our study, miR-451a successfully promoted the M1-to-M2 polarization of macrophages through the regulation of MIF. RT-qPCR results confirmed that the expression of MIF was upregulated by LPS and IFN-γ but downregulated after overexpression of miR-451a, and the expression of MIF also increased after inhibition of miR-451a expression. The experimental results show that MIF expression decreases after ISO-1 treatment and promotes the polarization of M1 macrophages to M2 macrophages. These data show that miR-451a enriched in ADSC-Exos promotes the M1-to-M2 conversion of macrophages by downregulating MIF expression. However, it is necessary to further study the specific mechanism of this process to guide follow-up clinical application.

## Conclusion

In conclusion, our experimental results show that ADSC-Exos can effectively promote bone healing and that mir-451a is enriched in extracted exosomes, which can inhibit inflammation and promote the polarization of M1 macrophages to M2 macrophages by targeting MIF. Compared with the cell therapy, exosomes are relatively easy to obtain and have low immunogenicity, so they have broad prospects in future applications. ADSC-Exos loaded in GNPs hydrogel can reach their target locations accurately to exert their effects. GNPs and exosomes cooperate with each other to provide physical and biological support at bone defects, which provides a new strategy for promoting bone healing.

## Supplementary Information

**Additional file 1: Table S1.** Primer sequences for RT-qPCR.

**Additional file 2: Figure S1.** Biosafety testing of GNPs and GNP-Exos.

## Data Availability

The datasets used and analysed during the current study are available from the corresponding author on reasonable request.

## Abbreviations

ADSC: Adipose-derived stem cells
ADSC-Exo: Exosomes from adipose-derived stem cells
MSC: Mesenchymal stem cell
GNP: Gelatine nanoparticle
GNP-Exo: Exosome-loaded gelatine nanoparticle
CM: Conditioned medium
Exos: Exosomes
miRNA: MicroRNA
BTE: Bone tissue engineering
iNOS: Myo-inositol-1-phosphate synthase
IL-10: Interleukin-10
CXCL-10: C-X-C motif chemokine ligand 10
TNF-α: Tumour necrosis factor alpha, matrix metalloproteinase-1
Arg-1: Arginase-1
miR-451a: MicroRNA-451a
MIF: Macrophage migration inhibitory factor
RT-qPCR: Reverse transcription-quantitative polymerase chain reaction
TEM: Transmission electron microscopy
FE-SEM: Field-emission scanning electron microscopy
LSCM: Laser confocal microscopy

## References

[1] Talevski J, Sanders KM, Busija L, Beauchamp A, Duque G, Borgström F (2021). Health service use pathways associated with recovery of quality of life at 12-months for individual fracture sites: analyses of the International Costs and Utilities Related to Osteoporotic fractures Study (ICUROS). Bone.

[2] Baldwin P, Li DJ, Auston DA, Mir HS, Yoon RS, Koval KJ (2019). Autograft, allograft, and bone graft substitutes: clinical evidence and indications for use in the setting of orthopaedic trauma surgery. J Orthop Trauma.

[3] Likhterov I, Roche AM, Urken ML (2019). Contemporary osseous reconstruction of the mandible and the maxilla. Oral Maxillofac Surg Clin N Am.

[4] Shegarfi H, Reikeras O (2009). Review article: bone transplantation and immune response. J Orthop Surg (Hong Kong).

[5] Agarwal R, García AJ (2015). Biomaterial strategies for engineering implants for enhanced osseointegration and bone repair. Adv Drug Deliv Rev.

[6] Kim HD, Amirthalingam S, Kim SL, Lee SS, Rangasamy J, Hwang NS (2017). Biomimetic materials and fabrication approaches for bone tissue engineering. Adv Healthc Mater.

[7] Park JY, Park SH, Kim MG, Park S-H, Yoo TH, Kim MS (2018). Biomimetic scaffolds for bone tissue engineering. Adv Exp Med Biol.

[8] Qu D, Mosher CZ, Boushell MK, Lu HH (2015). Engineering complex orthopaedic tissues via strategic biomimicry. Ann Biomed Eng.

[9] Walmsley GG, McArdle A, Tevlin R, Momeni A, Atashroo D, Hu MS (2015). Nanotechnology in bone tissue engineering. Nanomedicine.

[10] Saiz E, Zimmermann EA, Lee JS, Wegst UGK, Tomsia AP (2013). Perspectives on the role of nanotechnology in bone tissue engineering. Dent Mater.

[11] Terashima A, Takayanagi H (2019). The role of bone cells in immune regulation during the course of infection. Semin Immunopathol.

[12] Guder C, Gravius S, Burger C, Wirtz DC, Schildberg FA (2020). Osteoimmunology: a current update of the interplay between bone and the immune system. Front Immunol.

[13] Loi F, Córdova LA, Pajarinen J, Lin TH, Yao Z, Goodman SB (2016). Inflammation, fracture and bone repair. Bone.

[14] Bozec A, Soulat D (2017). Latest perspectives on macrophages in bone homeostasis. Pflug Arch.

[15] Sinder BP, Pettit AR, McCauley LK (2015). Macrophages: their emerging roles in bone. J Bone Miner Res.

[16] Spiller KL, Nassiri S, Witherel CE, Anfang RR, Ng J, Nakazawa KR (2015). Sequential delivery of immunomodulatory cytokines to facilitate the M1-to-M2 transition of macrophages and enhance vascularization of bone scaffolds. Biomaterials.

[17] Pajarinen J, Lin T, Gibon E, Kohno Y, Maruyama M, Nathan K (2019). Mesenchymal stem cell-macrophage crosstalk and bone healing. Biomaterials.

[18] Wasnik S, Rundle CH, Baylink DJ, Yazdi MS, Carreon EE, Xu Y (2018). 1,25-Dihydroxyvitamin D suppresses M1 macrophages and promotes M2 differentiation at bone injury sites. JCI Insight.

[19] Zhu G, Zhang T, Chen M, Yao K, Huang X, Zhang B (2021). Bone physiological microenvironment and healing mechanism: basis for future bone-tissue engineering scaffolds. Bioact Mater.

[20] Wubneh A, Tsekoura EK, Ayranci C, Uludağ H (2018). Current state of fabrication technologies and materials for bone tissue engineering. Acta Biomater.

[21] Sears NA, Seshadri DR, Dhavalikar PS, Cosgriff-Hernandez E (2016). A review of three-dimensional printing in tissue engineering. Tissue Eng Part B Rev.

[22] Kim H, Hyun MR, Kim SW (2019). The effect of adipose-derived stem cells on wound healing: comparison of methods of application. Stem Cells Int.

[23] Yoshida Y, Matsubara H, Fang X, Hayashi K, Nomura I, Ugaji S (2019). Adipose-derived stem cell sheets accelerate bone healing in rat femoral defects. PLoS ONE.

[24] Na YK, Ban J-J, Lee M, Im W, Kim M (2017). Wound healing potential of adipose tissue stem cell extract. Biochem Biophys Res Commun.

[25] Chen G, Jin Y, Shi X, Qiu Y, Zhang Y, Cheng M (2015). Adipose-derived stem cell-based treatment for acute liver failure. Stem Cell Res Ther.

[26] Hoffman JM, Sideri A, Ruiz JJ, Stavrakis D, Shih DQ, Turner JR (2018). Mesenteric adipose-derived stromal cells from Crohn's disease patients induce protective effects in colonic epithelial cells and mice with colitis. Cell Mol Gastroenterol Hepatol.

[27] Kruger MJ, Conradie MM, Conradie M, van de Vyver M (2018). ADSC-conditioned media elicit an ex vivo anti-inflammatory macrophage response. J Mol Endocrinol.

[28] López-Díaz de Cerio A, Perez-Estenaga I, Inoges S, Abizanda G, Gavira JJ, Larequi E (2021). Preclinical evaluation of the safety and immunological action of allogeneic ADSC-collagen scaffolds in the treatment of chronic ischemic cardiomyopathy. Pharmaceutics.

[29] Shukla L, Yuan Y, Shayan R, Greening DW, Karnezis T (2020). Fat therapeutics: the clinical capacity of adipose-derived stem cells and exosomes for human disease and tissue regeneration. Front Pharmacol.

[30] Cai Y, Li J, Jia C, He Y, Deng C (2020). Therapeutic applications of adipose cell-free derivatives: a review. Stem Cell Res Ther.

[31] Kong Y, Ma B, Liu F, Chen D, Zhang S, Duan J (2019). Cellular stemness maintenance of human adipose-derived stem cells on ZnO nanorod arrays. Small.

[32] Tuin SA, Pourdeyhimi B, Loboa EG (2016). Fabrication of novel high surface area mushroom gilled fibers and their effects on human adipose derived stem cells under pulsatile fluid flow for tissue engineering applications. Acta Biomater.

[33] Park HJ, Yu SJ, Yang K, Jin Y, Cho AN, Kim J (2014). Paper-based bioactive scaffolds for stem cell-mediated bone tissue engineering. Biomaterials.

[34] Seong JM, Kim B-C, Park J-H, Kwon IK, Mantalaris A, Hwang Y-S (2010). Stem cells in bone tissue engineering. Biomed Mater.

[35] Moreno Madrid AP, Vrech SM, Sanchez MA, Rodriguez AP (2019). Advances in additive manufacturing for bone tissue engineering scaffolds. Mater Sci Eng C Mater Biol Appl.

[36] Sato Y, Bando H, Di Piazza M, Gowing G, Herberts C, Jackman S (2019). Tumorigenicity assessment of cell therapy products: the need for global consensus and points to consider. Cytotherapy.

[37] Nirwan RS, Albini TA, Sridhar J, Flynn HW, Kuriyan AE (2019). Assessing "cell therapy" clinics offering treatments of ocular conditions using direct-to-consumer marketing websites in the United States. Ophthalmology.

[38] Ha DH, Kim H-K, Lee J, Kwon HH, Park G-H, Yang SH (2020). Mesenchymal stem/stromal cell-derived exosomes for immunomodulatory therapeutics and skin regeneration. Cells.

[39] Wu P, Zhang B, Shi H, Qian H, Xu W (2018). MSC-exosome: a novel cell-free therapy for cutaneous regeneration. Cytotherapy.

[40] Bucan V, Vaslaitis D, Peck C-T, Strauß S, Vogt PM, Radtke C (2019). Effect of exosomes from rat adipose-derived mesenchymal stem cells on neurite outgrowth and sciatic nerve regeneration after crush injury. Mol Neurobiol.

[41] Xiong M, Zhang Q, Hu W, Zhao C, Lv W, Yi Y (2020). Exosomes from adipose-derived stem cells: the emerging roles and applications in tissue regeneration of plastic and cosmetic surgery. Front Cell Dev Biol.

[42] Rani S, Ryan AE, Griffin MD, Ritter T (2015). Mesenchymal stem cell-derived extracellular vesicles: toward cell-free therapeutic applications. Mol Ther.

[43] Yamashita T, Takahashi Y, Takakura Y (2018). Possibility of exosome-based therapeutics and challenges in production of exosomes eligible for therapeutic application. Biol Pharm Bull.

[44] Jafari D, Malih S, Eini M, Jafari R, Gholipourmalekabadi M, Sadeghizadeh M, Samadikuchaksaraei A (2020). Improvement, scaling-up, and downstream analysis of exosome production. Crit Rev Biotechnol.

[45] Chang CL, Sung PH, Chen KH (2018). Adipose-derived mesenchymal stem cell-derived exosomes alleviate overwhelming systemic inflammatory reaction and organ damage and improve outcome in rat sepsis syndrome. Am J Transl Res.

[46] Blazquez R, Sanchez-Margallo FM, de La Rosa O, Dalemans W, Alvarez V, Tarazona R, Casado JG (2014). Immunomodulatory potential of human adipose mesenchymal stem cells derived exosomes on in vitro stimulated T cells. Front Immunol..

[47] Zhao H, Shang Q, Pan Z, Bai Y, Li Z, Zhang H (2018). Exosomes from adipose-derived stem cells attenuate adipose inflammation and obesity through polarizing M2 macrophages and beiging in white adipose tissue. Diabetes.

[48] Cho BS, Kim JO, Ha DH, Yi YW (2018). Exosomes derived from human adipose tissue-derived mesenchymal stem cells alleviate atopic dermatitis. Stem Cell Res Ther.

[49] Al-Sowayan B, Alammari F, Alshareeda A (2020). Preparing the bone tissue regeneration ground by exosomes: from diagnosis to therapy. Molecules.

[50] Zhu L, Feng X, Yang S, Wang J, Pan Y, Ding J (2021). Colorimetric detection of immunomagnetically captured rare number CTCs using mDNA-wrapped single-walled carbon nanotubes. Biosens Bioelectron.

[51] Lutz-Bueno V, Bolisetty S, Azzari P, Handschin S, Mezzenga R (2020). Self-winding gelatin-amyloid wires for soft actuators and sensors. Adv Mater.

[52] Wang H, Heilshorn SC (2015). Adaptable hydrogel networks with reversible linkages for tissue engineering. Adv Mater.

[53] Wang H, Hansen MB, Löwik DWPM, van Hest JCM, Li Y, Jansen JA, Leeuwenburgh SCG (2011). Oppositely charged gelatin nanospheres as building blocks for injectable and biodegradable gels. Adv Mater.

[54] TabatabaeiQomi R, Sheykhhasan M (2017). Adipose-derived stromal cell in regenerative medicine: a review. World J Stem Cells.

[55] Mu Z, Chen K, Yuan S, Li Y, Huang Y, Wang C (2020). Gelatin nanoparticle-injectable platelet-rich fibrin double network hydrogels with local adaptability and bioactivity for enhanced osteogenesis. Adv Healthc Mater.

[56] Kasuya S, Kato-Kogoe N, Omori M, Yamamoto K, Taguchi S, Fujita H (2018). New bone formation process using bio-oss and collagen membrane for rat calvarial bone defect: histological observation. Implant Dent.

[57] Chang CL, Sung PH, Chen KH, Shao PL, Yang CC, Cheng BC (2018). Adipose-derived mesenchymal stem cell-derived exosomes alleviate overwhelming systemic inflammatory reaction and organ damage and improve outcome in rat sepsis syndrome. Am J Transl Res.

[58] Seo Y, Kim HS, Hong IS (2019). Stem cell-derived extracellular vesicles as immunomodulatory therapeutics. Stem Cells Int.

[59] Cargnoni A, Papait A, Masserdotti A, Pasotti A, Stefani FR, Silini AR, Parolini O (2021). Extracellular vesicles from perinatal cells for anti-inflammatory therapy. Front Bioeng Biotechnol.

[60] Deng S, Zhou X, Ge Z, Song Y, Wang H, Liu X, Zhang D (2019). Exosomes from adipose-derived mesenchymal stem cells ameliorate cardiac damage after myocardial infarction by activating S1P/SK1/S1PR1 signaling and promoting macrophage M2 polarization. Int J Biochem Cell Biol.

[61] Yu X, Odenthal M, Fries JWU (2016). Exosomes as miRNA carriers: formation-function-future. Int J Mol Sci.

[62] Lu Y, Wen H, Huang J, Liao P, Liao H, Tu J, Zeng Y (2020). Extracellular vesicle-enclosed miR-486-5p mediates wound healing with adipose-derived stem cells by promoting angiogenesis. J Cell Mol Med.

[63] Zhao J, Li X, Hu J, Chen F, Qiao S, Sun X (2019). Mesenchymal stromal cell-derived exosomes attenuate myocardial ischaemia-reperfusion injury through miR-182-regulated macrophage polarization. Cardiovasc Res.

[64] Su Z, Ni L, Yu W, Yu Z, Chen D, Zhang E (2015). MicroRNA-451a is associated with cell proliferation, migration and apoptosis in renal cell carcinoma. Mol Med Rep.

[65] Babapoor S, Fleming E, Wu R, Dadras SS (2014). A novel miR-451a isomiR, associated with amelanotypic phenotype, acts as a tumor suppressor in melanoma by retarding cell migration and invasion. PLoS ONE.

[66] Xie J, Hu X, Yi C, Hu G, Zhou X, Jiang H (2016). MicroRNA-451 protects against cardiomyocyte anoxia/reoxygenation injury by inhibiting high mobility group box 1 expression. Mol Med Rep.

[67] Graham A, Falcone T, Nothnick WB (2015). The expression of microRNA-451 in human endometriotic lesions is inversely related to that of macrophage migration inhibitory factor (MIF) and regulates MIF expression and modulation of epithelial cell survival. Hum Reprod.

[68] Li Q, Li Y, Zhang D, Gao H, Gao X (2019). Downregulation of microRNA-451 improves cell migration, invasion and tube formation in hypoxia-treated HUVECs by targeting MIF. Mol Med Rep.

[69] Barbosa de Souza Rizzo M, Brasilino de Carvalho M, Kim EJ, Rendon BE, Noe JT, Darlene Wise A, Mitchell RA (2018). Oral squamous carcinoma cells promote macrophage polarization in an MIF-dependent manner. QJM..

[70] Zhang Z, Zhang R, Li L, Zhu L, Gao S, Lu Q (2018). Macrophage migration inhibitory factor (MIF) inhibitor, Z-590 suppresses cartilage destruction in adjuvant-induced arthritis via inhibition of macrophage inflammatory activation. Immunopharmacol Immunotoxicol.

