# Supplementary material for: Exosomes from adipose-derived stem cells regulate M1/M2 macrophage phenotypic polarization to promote bone healing via miR-451a/MIF
Source: Stem Cell Res Ther. 2022 Apr 8;13:149. doi: 10.1186/s13287-022-02823-1 (PMC8994256; doi:10.1186/s13287-022-02823-1)
Supplement: Supplementary file 1 — Additional file 1: Table S1. Primer sequences for RT-qPCR. [file 13287_2022_2823_MOESM1_ESM.docx]

**Supplementary Table S1** Primer sequences for RT-qPCR

| Gene | Forward primer (5′–3′） | Reverse primer (5′–3′) |
| --- | --- | --- |
| CD86 | GTTTCATTCCCTGATGTTACGAG | GAGAAAGGTGAAGATAAAAGCCG |
| CD206 | TGATACCTGCGACAGTAAACGA | CTTGCAGTATGTCTCCGCTTC |
| CD163 | TCGCTCATCCCGTCAGTCA | CCGCTGTCTCTGTCTTCGCT |
| iNOS | CAGGACTCACAGCCTTTGGAC | TGGATGTCGGACTTTGTAGATTC |
| IL-10 | GAGAGAAGCTGAAGACCCTCTG | TCATTCATGGCCTTGTAGACAC |
| CXCL-10 | CCGGAATTCGAGCCTACAGCAGAGGAACC | CCGCTCGAGTTTGCTCCCCTCTGGTTTTA |
| TNF-α | GCGACGTGGAACTGGCAGAAG | GCCACAAGCAGGAATGAGAAGAGG |
| Arg-1 | TAACCTTGGCTTGCTTCGGAACTC | GGCGCATTCACAGTCACTTAGG |
| miR-451a | GAAACCGTTACCATTACTGAG | -- |
| MIF | CCGGACAGGGTCTACATCAAC | TTAGGCGAAGGTGGAGTTGTT |
| U6 | CGCTTCGGCAGCACATATAC | AAATATGGAACGCTTCACGA |
| β-actin | AGAAAATCTGGCACCACACCT | GATAGCACAGCCTGGATAGCA |

iNOS myo-inositol-1-phosphate synthase,IL-10 Interleukin-10, CXCL-10 C-X-C motif chemokine ligand 10, TNF-α tumor necrosis factor alpha,matrix metalloproteinase-1, Arg-1 arginase 1, miR-451a microRNA-451a, MIF macrophage migration inhibitory factor,RT-qPCR Reverse transcription-quantitative polymerase chain reaction
