# Supplementary figures and images for: Exosomes from adipose-derived stem cells regulate M1/M2 macrophage phenotypic polarization to promote bone healing via miR-451a/MIF
Source: Stem Cell Res Ther. 2022 Apr 8;13:149. doi: 10.1186/s13287-022-02823-1 (PMC8994256; doi:10.1186/s13287-022-02823-1)

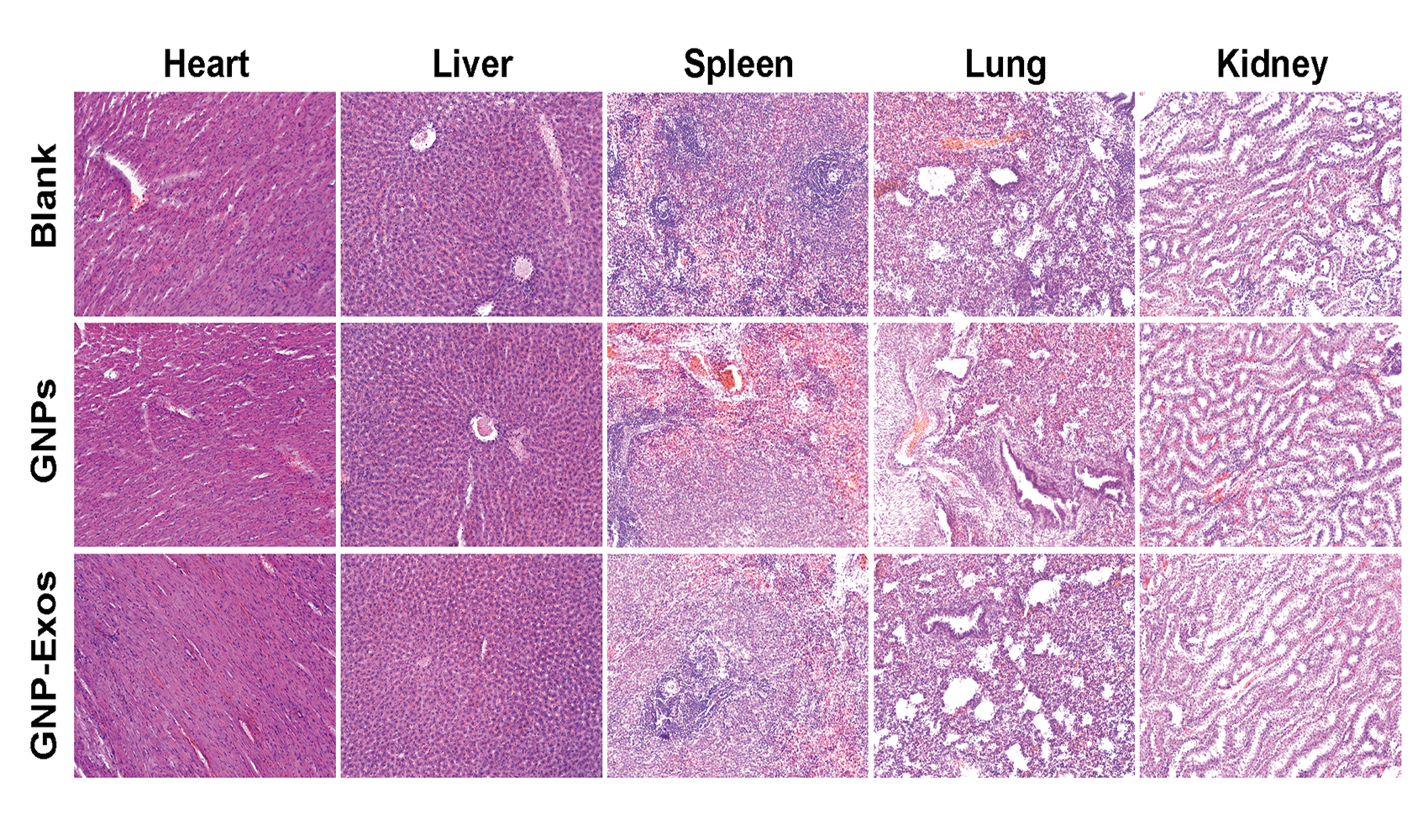

Supplement: Supplementary file 2 — Additional file 2: Figure S1. Biosafety testing of GNPs and GNP-Exos. [file 13287_2022_2823_MOESM2_ESM.tif]
